# Metabolite 4-hydroxybenzoic acid, modulated by cigarette smoke exposure, functions as a protective factor in dextran sodium sulfate-induced colitis

**DOI:** 10.1007/s00535-026-02484-4

**Published:** 2026-07-31

**Authors:** Masahiro Kitabatake, Tatsuki Nishioka, Atsushi Hara, Kaito Yasuike, Yumiko Nakanishi, Eiji Miyauchi, Noriko Ouji-Sageshima, Hiroshi Ohno, Toshihiro Ito

**Affiliations:** 1 Department of Immunology, Nara Medical University, 840 Shijo-cho, Kashihara, Nara 634-8521 Japan; 2 Laboratory for Intestinal Ecosystem, RIKEN Center for Integrative Medical Sciences, 1-7-22 Suehiro-cho, Tsurumi-ku, Yokohama, Kanagawa 230-0045 Japan; 3 Present Address: RIKEN GENESIS CO., LTD, 1-2-2 Osaki, Shinagawa-ku, Tokyo, 141-0032 Japan; 4 Present Address: Institute for Molecular and Cellular Regulation, Gunma University, 3-39-15 Showa-machi, Maebashi, Gunma 371-8512 Japan

**Keywords:** Inflammatory bowel diseases, Smoking, Murine colitis models, Microbiota, 4-Hydroxybenzoic acid

## Abstract

**Background:**

Inflammatory bowel disease (IBD) is characterized by chronic gastrointestinal inflammation and comprises ulcerative colitis (UC) and Crohn’s disease (CD). Several cohort studies have shown that cigarette smoking (CS) is a risk factor for CD, but it has protective potential for UC. Although CS has been shown to cause different effects on UC and CD, the underlying mechanisms have not been fully determined.

**Methods:**

Colitis was induced in CS-exposed mice using two different chemical-induced models: dextran sodium sulfate (DSS) and 2,4,6-trinitrobenzene sulfonic acid (TNBS). We examined pathology of the colon, effector lymphocytes, composition of the microbiota, and intestinal metabolites. We also examined the effect of an intestinal metabolite, 4-hydroxybenzoic acid (4-HBA), on DSS- and TNBS-induced colitis.

**Results:**

CS exposure attenuated the pathogenesis of DSS-induced colitis by an increase in regulatory T cells (Tregs) accompanied by an increase in short-chain fatty acid production. By contrast, CS exacerbated TNBS-induced colitis by an increase in effector T cells and the abundance of *Enterobacteriaceae*, which were correlated with the pathogenesis of IBD. The administration of 4-HBA, which was remarkably increased by CS exposure in the DSS-induced colitis model, suppressed the colonic inflammation accompanying the induction of Tregs and suppression of effector T cells, but exerted no apparent protective effect against TNBS-induced colitis model.

**Conclusion:**

CS may regulate colonic inflammation by altering the composition of the microbiota, intestinal metabolites, and immune responses. Additionally, mechanistic findings identified 4-HBA as a potential therapeutic target and provided insights into the development of new therapeutic approaches for UC.

**Supplementary Information:**

The online version contains supplementary material available at https://doi.org/10.1007/s00535-026-02484-4.

## Introduction

Inflammatory bowel disease (IBD) is characterized by chronic inflammation of the gastrointestinal tract with repetition of remission and relapse [1, 2 ]. The number of patients with IBD has been increasing worldwide in recent years. IBD can be divided into two types: ulcerative colitis (UC) and Crohn’s disease (CD). UC causes inflammation mainly in the mucosal and submucosal layers of the large intestine continuously from the rectum, while CD causes transmural inflammation discontinuously in the whole gastrointestinal tract. The etiology of both diseases is still unclear. However, many studies have indicated that an imbalance and/or deficiency of an individual’s genetic factors, environmental factors, intestinal bacteria, and mucosal immune system lead to the development of diseases [3–5]. An example of this situation is that aberrant activation of T helper type (Th) 1 cells and Th17 cells or deficiency of suppression mechanisms, including interleukin (IL-10) and regulatory T cells (Tregs), are associated with IBD. Additionally, approximately 100 trillion bacteria of 500–1000 species co-existing in the large intestine mutually regulate the mucosal immune system, and an imbalance of intestinal bacterial composition, called “dysbiosis”, has been observed in patients with IBD [6–8].

Cigarette smoking (CS) is a global public health issue, with approximately one third of the adult population estimated to be smokers [9]. CS is a well-known major risk factor for various diseases, such as cancer, respiratory disease, cardiovascular disease, and diabetes. Several cohort studies have shown that CS increases the incidence and severity of CD [10, 11 ]. Smokers have an odds ratio of 2.0 for developing CD compared with non-smokers, and this risk increases 3.4-fold with smoking [12]. By contrast, CS is associated with a lower incidence of UC, and cessation of smoking can worsen symptoms in patients with UC. Current smokers have an odds ratio of 0.41 for developing UC compared with non-smokers [12]. These epidemiological data suggest that CS is a risk factor for the onset and exacerbation of CD, but a protective factor for UC. Although CS has been shown to cause conflicting effects on UC and CD, the underlying mechanisms have not been fully determined.

In this study, we investigated the effect of CS on the pathogenesis of two types of murine colitis models using dextran sodium sulfate (DSS) and 2,4,6-trinitrobenzene sulfonic acid (TNBS). DSS mainly induces inflammation in the superficial mucosa, similar to UC [13], and TNBS induces the Th1 cell-mediated response and transmural inflammation, which are features of CD [14]. We also analyzed the resulting changes in the intestinal effector T-cell population, gut microbiota, and intestinal metabolite composition. Furthermore, we examined the effect of an intestinal metabolite, 4-hydroxybenzoic acid (4-HBA), which is altered by CS exposure, on the colitis models.

## Methods

### Mice

Female C57BL/6N mice (7 weeks old) were purchased from CLEA Japan (Tokyo, Japan), and maintained under specific pathogen-free conditions at the Animal Research Center, Nara Medial University. All procedures were carried out in accordance with the regulations for animal care. The animal experimental protocols were approved by the Ethics Review Committee for Animal Experimentation of Nara Medical University (approval number: 12232), and all procedures were carried out according to the Policy on the Care and Use of Laboratory Animals, Nara Medical University. The details methods are provided in Supplemental information.

### CS exposure

Non-filtered cigarettes, containing nicotine 2.3 mg and tar 28 mg, were purchased from Japan Tabacco Inc. (Peace®; Tokyo, Japan). Mice were exposed to smoke from 20 cigarettes for 30 min, 6 days a week using an exposure apparatus (MIPS Inc., Osaka, Japan) as previously reported [15].

### Colitis models

DSS (molecular weight: 36,000–50,000 Da, colitis grade) was purchased from MP Biomedicals (Santa Ana, CA). At 2 weeks after initiating CS exposure, the mice were administrated 3% DSS in drinking water ad libitum for 5 days, followed by tap water for 2 days. Disease activity was evaluated using the disease activity index (DAI), as previously described [16]. In some experiments, 4-HBA (Sigma-Aldrich, St Louis, MO) was orally administered at 50 mg/kg in mice every other day from 7 days before starting DSS administration. In another model of colitis, following overnight fasting, mice were intrarectally administrated 3 mg of TNBS (Tokyo Chemical Industry, Tokyo, Japan) dissolved in 100 μl of 50% ethanol using a vinyl catheter that was inserted 3.5 cm from the anus under anesthetization with sodium pentobarbital (50 mg/kg; Fujifilm Wako, Osaka, Japan) or a combination of medetomidine (0.75 mg/kg, Nippon Zenyaku Kogyo, Fukushima, Japan), midazolam (4 mg/kg, Maruishi Pharmaceutical, Osaka Japan), and butorphanol (5 mg/kg, Meiji Animal Health, Tokyo, Japan). After TNBS injection, the mice were kept in the head down position for 5 min. In some experiments, 4-HBA (50 mg/kg) was orally administered every other day from 7 days before TNBS administration, followed by three additional doses after colitis induction. The large intestines were surgically excised, their length was measured, and then sections of rectal side were stained with hematoxylin and eosin (Sakura Finetek Japan Co., Ltd., Tokyo, Japan). The pathogenesis of colitis was assessed by two independent pathologists according to the histological score, as previously described [17]. Gene expression levels of the inflammatory cytokines, (*Il1b*, *Il6,* and *Tnf*) were analyzed by quantitative PCR.

### Flow cytometry

Lymphocytes from the lamina propria (LPLs) in the colon of colitis-induced mice were isolated by using the Lamina Propria Dissociation Kit with the gentleMACS dissociator (Miltenyi Biotec GmbH, Bergisch Gladbach, Germany). LPLs were stained with antibodies against surface and intracellular antigens, and proportions of Tregs, Th1, Th17 and IFN-γ^+^ CD8^+^ T cells were analyzed using the MACSQuant Analyzer (Miltenyi Biotec GmbH) or the Northern Lights spectral flow cytometer (Cytek Biosciences, Fremont, CA). Data were analyzed by FlowJo software (Tree Star, Ashland, OR), and gating strategies for the detection of these cell populations are shown in Supplementary Fig. 1.

### In vitro Treg differentiation

CD4^+^ T cells were isolated from mesenteric lymph nodes of C57BL/6N mice using a CD4^+^ T cell isolation kit and autoMACS separator (Miltenyi Biotec GmbH) according to the manufacturer’s instructions. The cells were stimulated with coated anti-CD3 mAb (Biolegend) and soluble factors, including anti-CD28 mAb (Biolegend), murine IL-2 (Peprotech, Cranbury, NJ), and human TGF-β (Peprotech), in the presence or absence of 4-HBA (100 μg/ml) for 48 h. After the stimulation, Treg differentiation was evaluated by measuring Foxp3 expression using flow cytometry and quantitative PCR, as described above.

### Microbiota analysis

Fecal samples were obtained from mice and then immediately frozen by dry ice. Extraction of bacterial DNA, sequencing, and software analysis were carried out by Morinaga Milk Industry (Shizuoka, Japan), as described previously [18]. Briefly, bacterial DNA was extracted from fecal samples, and then the V3–V4 region of the bacterial 16S ribosomal RNA gene was amplified by PCR. The PCR product was further amplified using the barcoded primers adapted for the Illumina MiSeq system (Illumina Inc., San Diego, CA). The pooled libraries from equal amounts of amplicons were sequenced by the Illumina Miseq system with the MiSeq Reagent Kit v3. Sequence data were analyzed using the QIIME software package version 1.8.0 (http://qiime.org/) and the Greengenes reference database (gg_13_5_99). Linear regression analysis was performed on the relative abundance of gut microbiota at the family level using the R package MaAsLin2.

### Metabolite analysis

An analysis of intestinal metabolites in feces was conducted as described elsewhere [19]. Fecal samples were extracted separately for the analysis of water-soluble metabolites and short-chain fatty acids (SCFAs). The extracts were subjected to derivatization and subsequently analyzed by gas chromatography–tandem mass spectrometry platform on a GCMS-TQ8030 (Shimadzu, Kyoto, Japan). The data were processed using LabSolution Insight (Shimadzu, Kyoto, Japan).

### Statistical analysis

Data are presented as the mean ± standard error of the mean (SEM) and are representative of at least two independent experiments. Student’s *t* test was used to perform statistical analyses of body weight changes, colon length, gene expression, metabolites levels, and lymphocyte populations between the control and CS-exposed group using GraphPad Prism 9 (GraphPad Software, San Deigo, CA). Statistical analysis of metabolites levels across all models was performed by one-way analysis of variance with Tukey’s post hoc test. For DAI and histological scores, the Mann–Whitney *U* test was used. Differences were considered statistically significant at *P* < 0.05.

## Results

### Varying effects of CS exposure on the pathogenesis of different murine colitis models

**Fig. 1:**
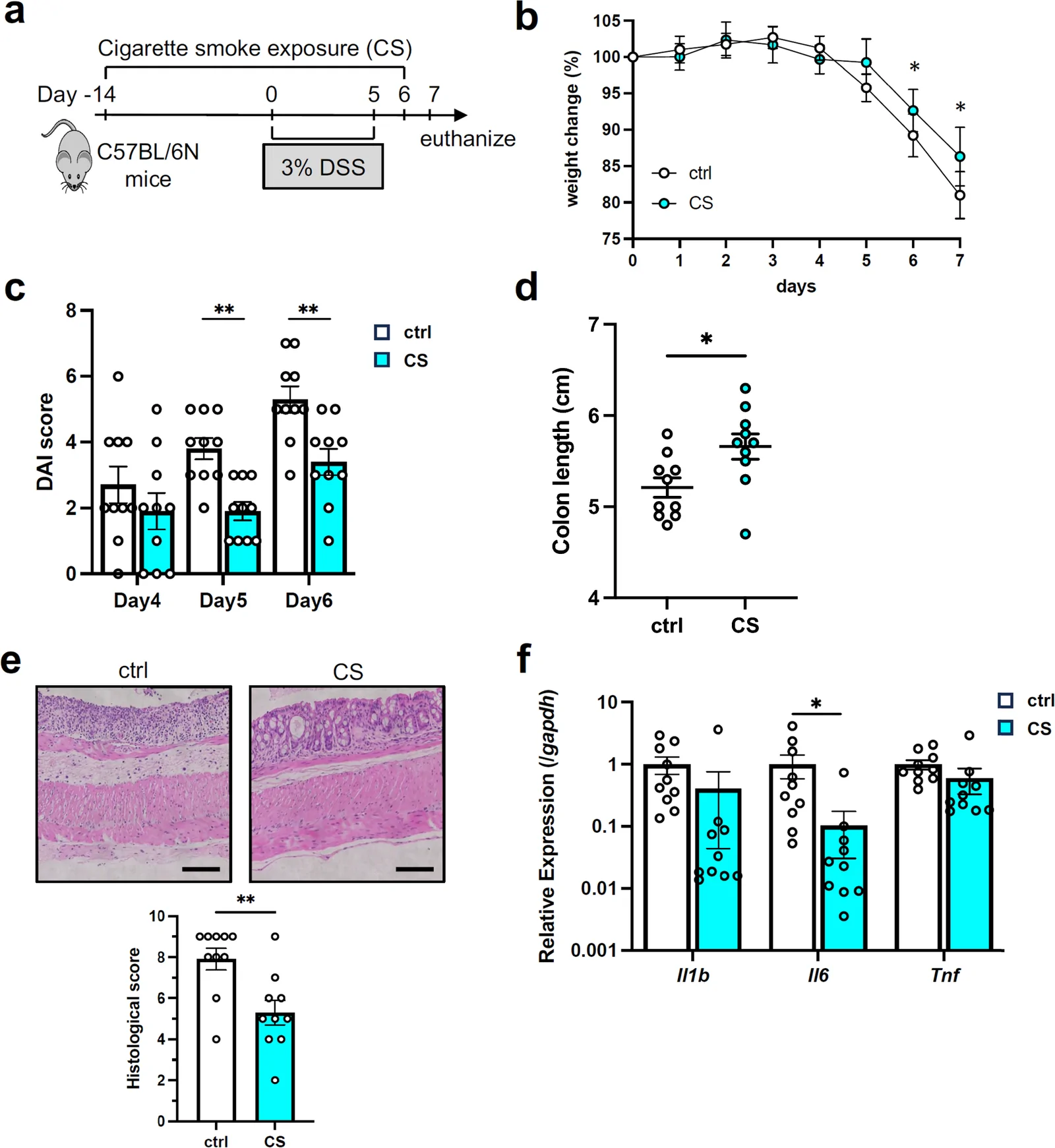
CS exposure ameliorates DSS-induced colitis. **a** Schematic model of the experimental design. C57BL/6N mice were exposed to CS for 2 weeks and then administered 3% DSS in drinking water for 5 days. **b** Change in body weight in the control and CS-exposed groups. **c** The disease activity index (DAI) was monitored from days 4 to 6 after DSS administration. **d** Colon length at day 7 after DSS administration. **e** The sections of proximal colon region were stained with hematoxylin and eosin. Representative images are shown in the upper panel. Histological scores are shown in the bottom panel. Bars 100 µm. **f** IL-1β, IL-6, and TNF-α gene expression in the colon. Data are shown as the mean ± SEM (*n* = 10 in each group). *P < 0.05, **P < 0.01

CS has varied effects on UC and CD [10]. We investigated the effect of CS on the pathogenesis of colitis using two types of murine colitis models. First, we exposed mice to CS for 2 weeks, and then colitis was induced by 3% DSS administration in drinking water for 5 days (Fig. 1a). Weight loss by DSS administration was reduced by CS exposure (Fig. 1b). The DAI was significantly lower from days 5 to 6 in CS-exposed mice than in control mice (day 5 *P* = 0.0012; day 6 *P* = 0.0053) (Fig. 1c). The colon length, generally shortened by inflammation, was longer in CS-exposed mice than in control mice (Fig. 1d). The histological analysis showed that the pathology of colitis, including distortion of the crypt architecture, hyperplasia, and infiltration of leucocytes, and the histological score were significantly improved by CS exposure (*P* = 0.0019) (Fig. 1e). Gene expression of inflammatory cytokine *Il6* was decreased in CS-exposed mice, which suggested that CS exposure attenuated the DSS-induced colitis (Fig. 1f).

**Fig. 2:**
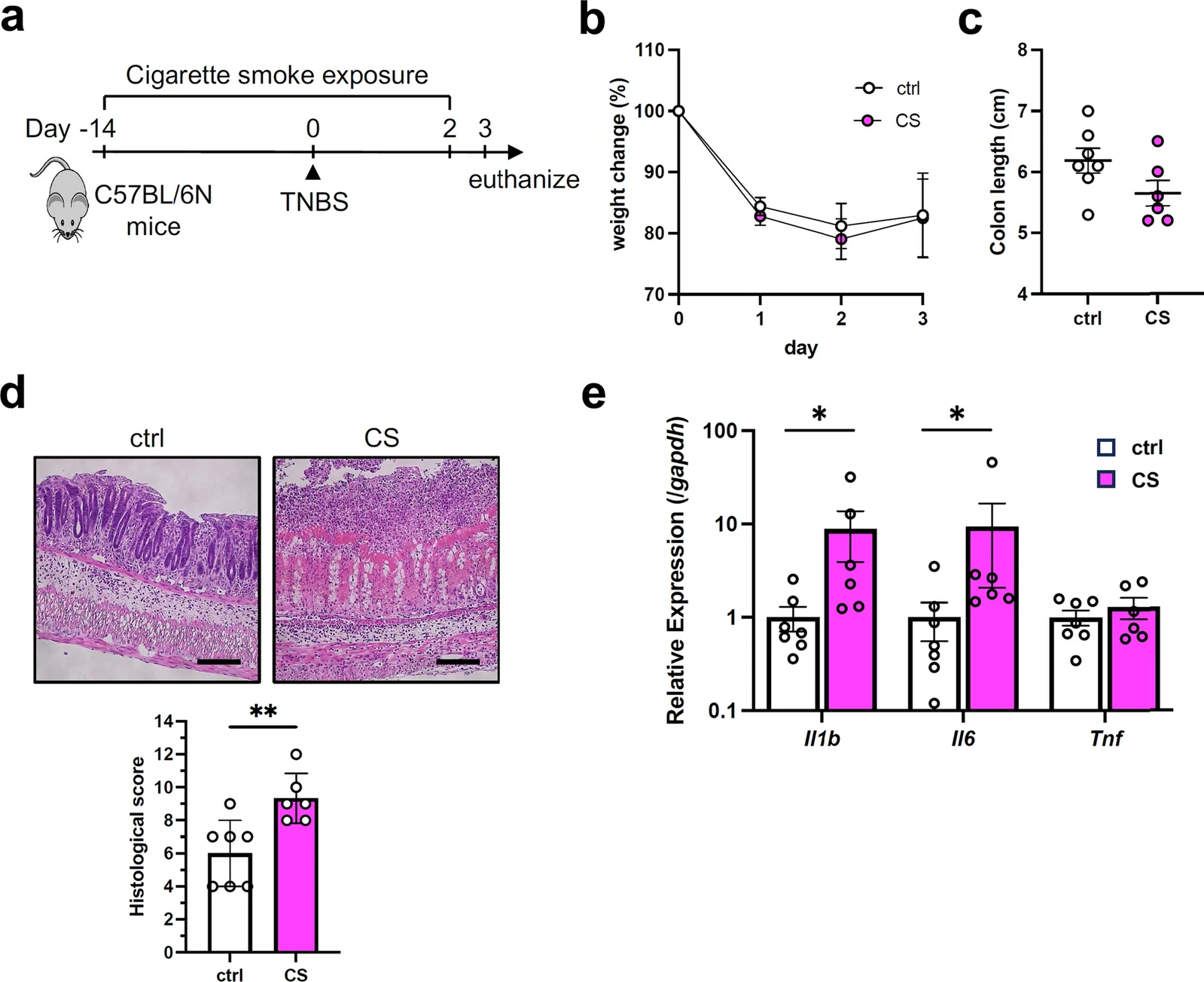
CS exposure exacerbates TNBS-induced colitis. **a** Schematic model of the experimental design. C57BL/6N mice were exposed to CS for 2 weeks and then 3 mg of TNBS in 50% ethanol was intrarectally administered. **b** Change in body weight in the control and CS-exposed groups. **c** Colon length at day 3 after TNBS administration. **d** Sections of the proximal colon region were stained with hematoxylin and eosin. Representative images are shown in the upper panel. Histological scores are shown in the bottom panel. Bars 100 µm. **e** IL-1β, IL-6, and TNF-α gene expression in the colon. Data are shown as the mean ± SEM (*n* = 6–7). *P < 0.05, **P < 0.01

We created another chemical-induced colitis model using rectal administration of TNBS, which induced transmural inflammation (Fig. 2a). Weight loss after TNBS administration was not significantly different between control and CS-exposed mice (Fig. 2b). The colon length tended to be shorter in CS-exposed mice than in control mice, but this was not significant (Fig. 2c). However, the histological analysis showed that colon inflammation and destruction of tissue architecture, such as shedding of epithelial cells, were exacerbated in CS-exposed mice (Histological score *P* = 0.0087) (Fig. 2d). Gene expression of inflammatory cytokines, including *Il1b* and *Il6,* was significantly increased in CS-exposed mice (Fig. 2e). These results suggested that CS exposure showed different effects in the DSS and TNBS colitis models.

### Effect of CS on effector T cells in the colitis models

**Fig. 3:**
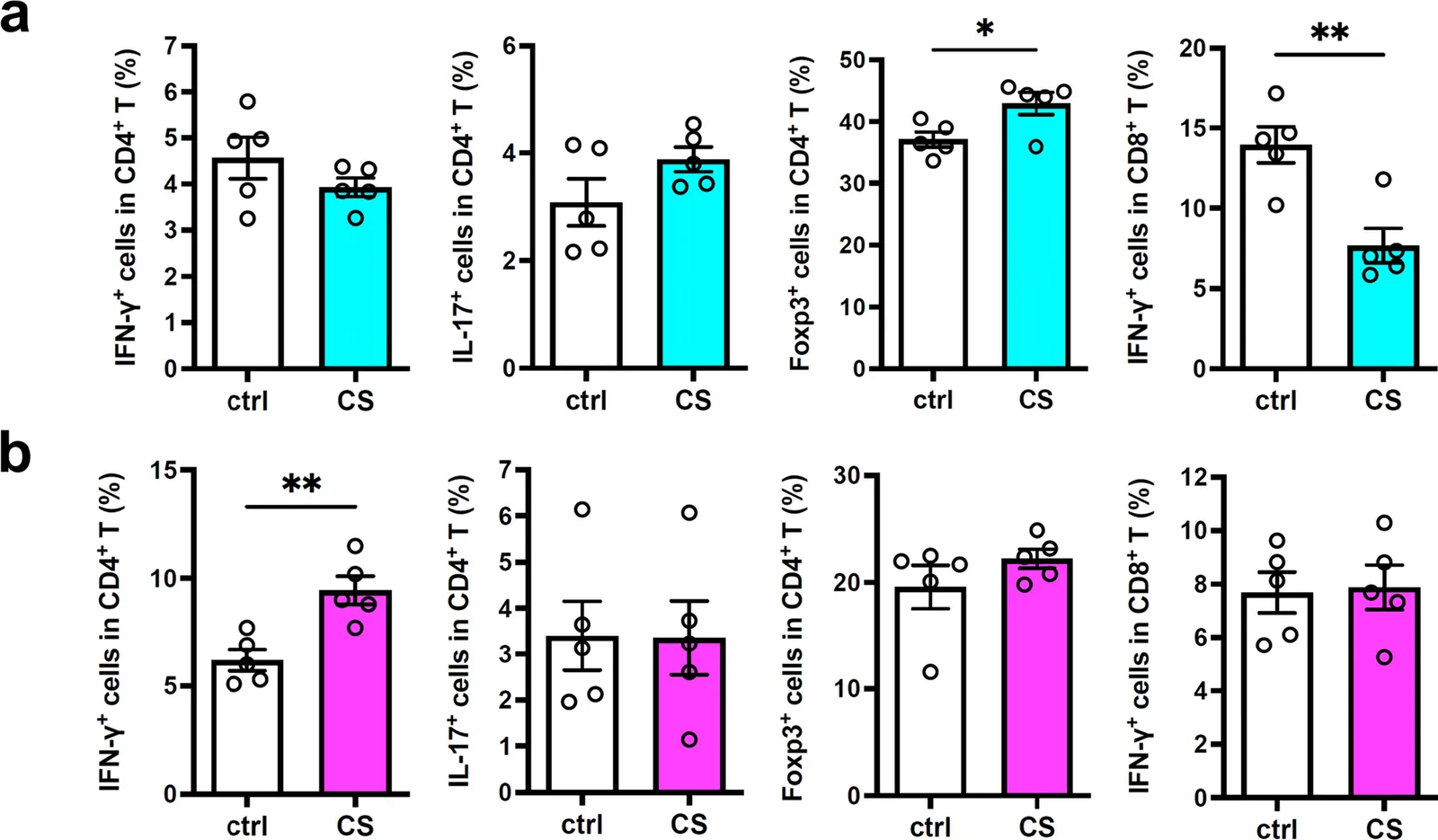
CS exposure affects the proportion of effector T cells in the DSS-induced and TNBS-induced colitis models. Lymphocytes from the lamina propria were isolated from the control and CS-exposed groups in the DSS-induced colitis model at day 7 (**a**) and TNBS-induced colitis model at day 3 (**b**). Proportions of Th1 cells (IFN-γ^+^ CD4^+^ T), Th17 cells (IL-17A^+^ CD4^+^ T), Tregs (Foxp3^+^ CD4^+^ T), and activated CD8^+^ T cells (IFN-γ^+^ CD8^+^ T) were analyzed by flow cytometry. Data are shown as the mean ± SEM (*n* = 5 in each group). *P < 0.05, **P < 0.01

Previous studies have reported that various types of effector T cells are involved in the pathogenesis of IBD [5]. Therefore, we investigated the effect of CS exposure on effector T cells in the colon lamina propria in colitis models. In the DSS-induced colitis model, the proportions of Th1 (IFN-γ^+^ CD4^+^ T) and Th17 (IL-17A^+^ CD4^+^ T) cells, which contribute to the pathogenesis of IBD, were similar in control and CS-exposed mice (Fig. 3a). However, the proportion of Foxp3^+^ CD4^+^ Tregs was significantly higher in CS-exposed mice than in control mice (*P* = 0.026). Interestingly, IFN-γ^+^-activated CD8^+^ T cells were significantly lower in CS-exposed mice than in control mice (*P* = 0.0037), which suggested that the CD8^+^ T cell-mediated inflammatory response was suppressed by CS exposure via the induction of Tregs. Conversely, CS exposure increased the proportion of Th1 cells in TNBS-induced colitis (*P* = 0.0041) (Fig. 3b). However, the proportions of Th17 cells, IFN-γ^+^-activated CD8^+^ T cells, and Tregs in CS-exposed mice were similar to those in control mice. These results suggest that CS exposure has different effects on the induction of effector T cells depending on the colitis model.

### Effect of CS on the microbiota in the colitis models

**Fig. 4:**
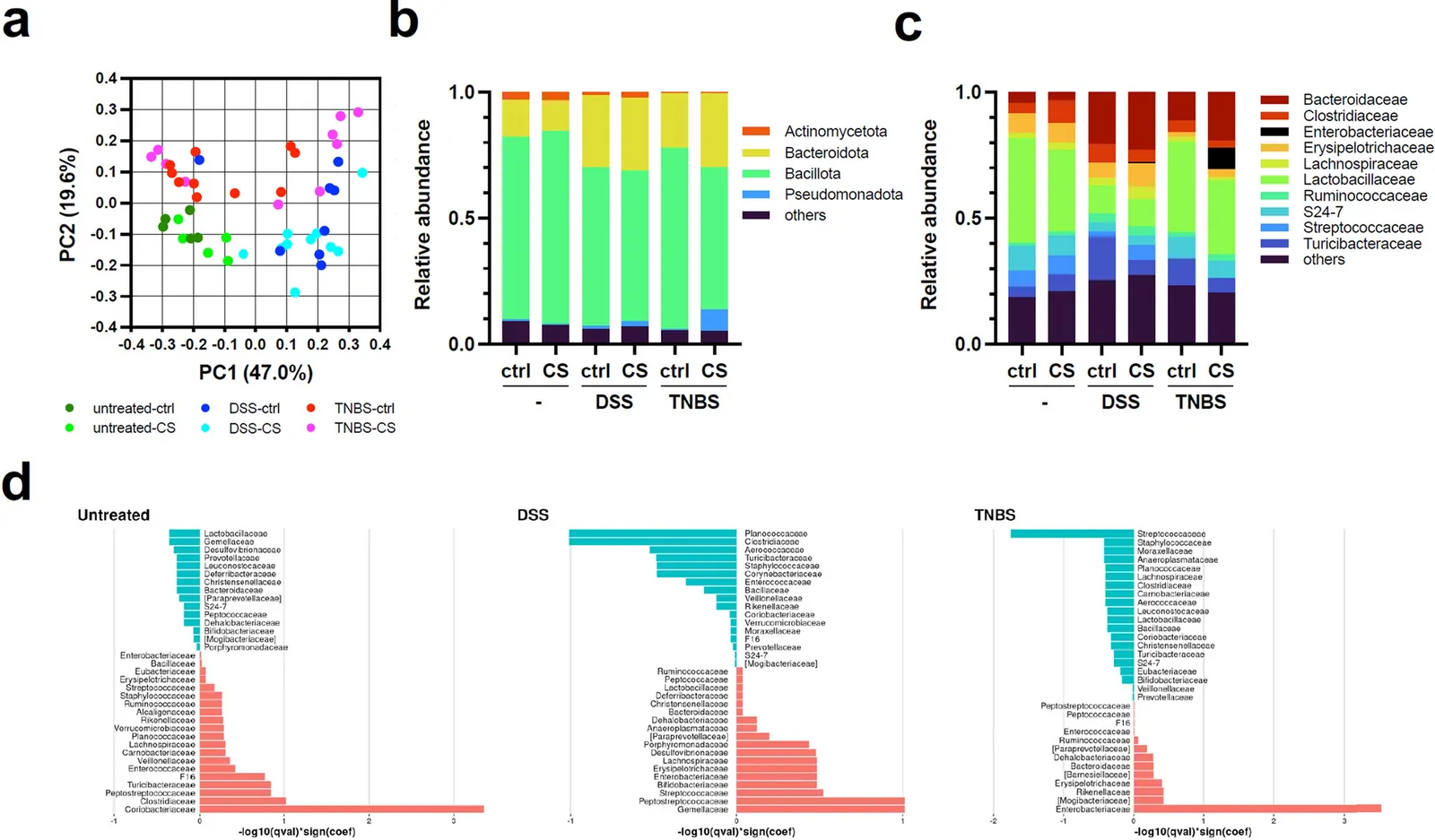
Different effects of CS exposure on the composition of the microbiota in DSS-induced and TNBS-induced colitis. The composition of fecal bacteria in the control and CS exposed-groups in untreated, DSS-treated (day 7), and TNBS-treated mice (day 3) was analyzed by 16S ribosomal RNA gene sequencing. **a** A principal coordinate analysis plot of bacterial composition. **b, c** Relative abundance of bacteria at the phylum with a presence greater than 1% (**b**), and the top 10 abundant families (**c**). **d** The difference at the family levels between control and CS-exposed group in untreated (left; *n* = 5 in each group), DSS-treated (middle; *n* = 8–10), and TNBS-treated mice (right; *n* = 10 in each group) analyzed by linear regression analysis.

We then assessed the effect of CS exposure on the composition of the fecal/gut microbiota. Fecal samples were collected and examined from control and CS-exposed groups under the following conditions: untreated, DSS-treated mice at day 7, and TNBS-treated mice at day 3. A principal coordinate analysis showed that the composition of the fecal microbiota changed by the induction of colitis (Fig. 4a). Consistent with this, the relative abundance of bacteria at both the phylum and family levels were altered in each colitis model (Fig. 4b, c). The α-diversity indices, the chao1 index and the Shannon index, which estimate the bacterial richness and accounts for richness and evenness, respectively, were not significantly altered among the untreated, DSS-treated, and TNBS-treated mice in the control group (Supplementary Fig. 2a). In the comparison between the control group and the CS-exposed group, these α-diversity indices in the CS-exposed group tended to be higher than in the control group in DSS-treated mice, but were lower in TNBS-treated mice (Supplementary Fig. 2a). In untreated mice, the bacterial composition at the phylum level remained largely unchanged between the control and CS-exposed groups (Fig. 4b). However, at the family level, the relative abundance of *Coriobacteriaceae* was significantly higher in the CS-exposed group compared with the control group (*P* = 0.0004) (Fig. 4c, d, Supplementary Fig. 2b). For DSS-induced colitis, the relative abundance of bacteria did not significantly change at the phylum and family levels. Interestingly, while the relative abundance of *Gemellaceae* and *Peptostreptococcaceae*, which are considered pathobionts, tend to be higher in the CS-exposed group, the relative abundance of *Bifidobacteriaceae* and *Lachnospiraceae*, which includes bacteria producing SCFAs, also tended to be higher in the CS-exposed group than in the control group (Supplementary Fig. 2b). At the phylum level in TNBS-treated mice, the relative abundance of Pseudomonadota (formerly Proteobacteria) was markedly higher in the CS-exposed group compared with the control group (*P* = 0.0056) (Fig. 4b). At the family level, the relative abundance of *Enterobacteriaceae*, which belongs to Pseudomonadota, was remarkably higher in the CS-exposed group than in control group (*P* = 0.0003) (Fig. 4c, d, Supplementary Fig. 2b). These results indicate that CS exposure dramatically changes the composition of the microbiota and is dependent on the colitis model.

### Effect of CS on intestinal metabolites in the colitis models

**Fig. 5:**
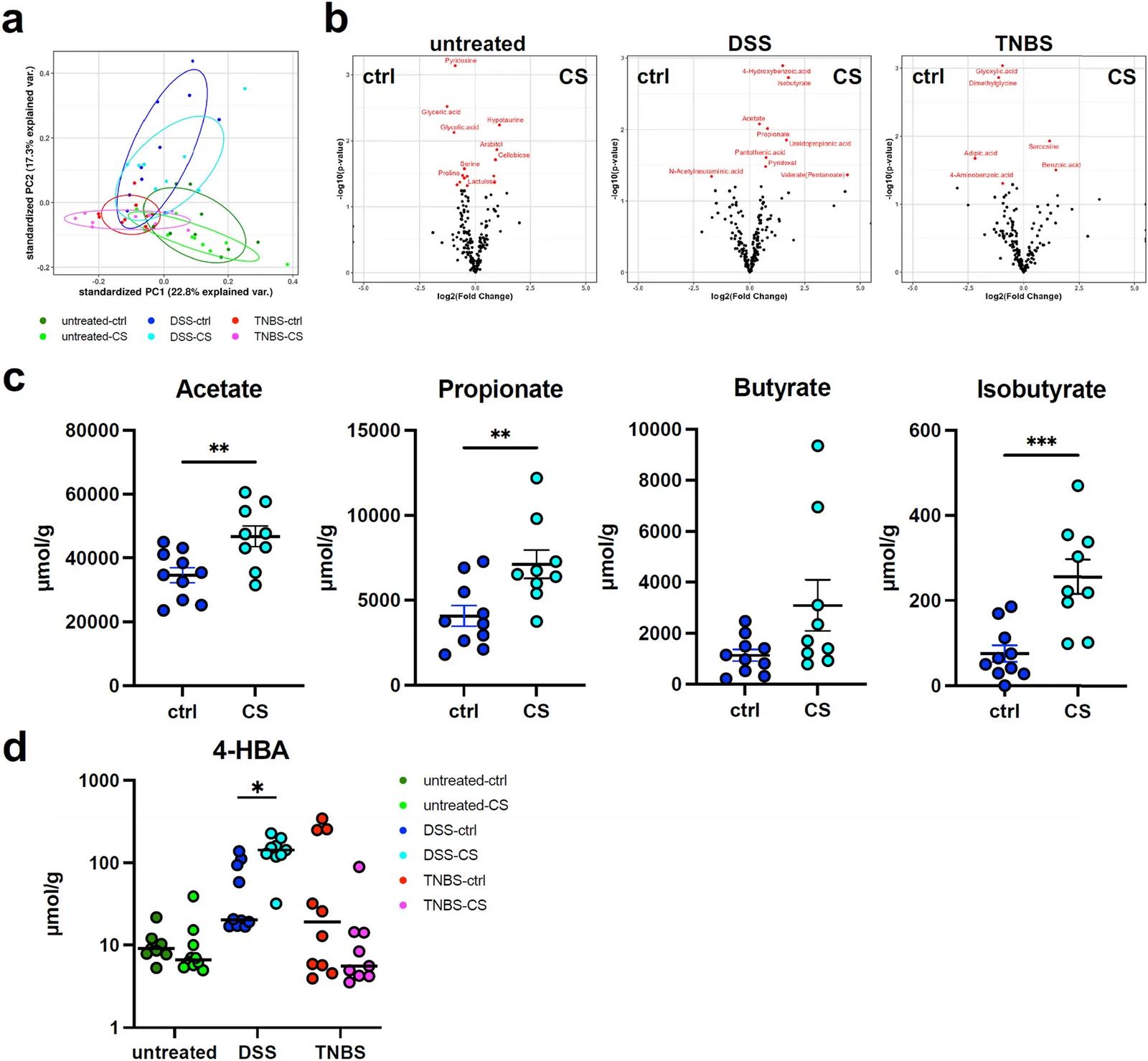
CS exposure affects the intestinal metabolites of the microbiota. The composition of intestinal metabolites in feces from the control and CS-exposed groups in untreated, DSS-treated (day 7), and TNBS-treated mice (day 3) was analyzed by gas chromatography–tandem mass spectrometry. **a** Principal component analysis plot of intestinal metabolites. **b** Volcano plots of the difference in each metabolite between the control and CS-exposed groups in untreated (left), DSS-treated (middle), and TNBS-treated mice (right). Each plot represents the average value of one metabolite from each group. **c** Concentrations of acetate, propionate, butyrate, and isobutyrate in the feces. **d** Concentrations of 4-HBA in the feces. Data are shown as the mean ± SEM with individual plots (*n* = 9–10). *P < 0.05, **P < 0.001

On the basis of our finding that the composition of the intestinal microbiota in colitis models was altered by CS exposure, we analyzed the effect of CS exposure on intestinal metabolites. Fecal samples were collected from control and CS-exposed groups under the following conditions: untreated, DSS-treated mice at day 7, and TNBS-treated mice at day 3. A total of 276 metabolites were analyzed. A principal component analysis showed that the composition of intestinal metabolites was changed in each colitis model (Fig. 5a). The top 50 metabolites that were differentially expressed across all samples and the top 25 metabolites with CS-related differences within individual models are shown in Supplementary Fig. 3a and b. In untreated mice, CS-exposed group showed higher levels of disaccharides, including lactulose and cellobiose, which are not hydrolyzed by host digestive enzymes, but are fermented by the gut microbiota and becoming a substrate for SCFAs production (Fig. 5b). In the DSS-induced colitis model, CS exposure significantly increased SCFAs, including acetate (*P* = 0.0068), propionate (*P* = 0.0080), and isobutyrate (*P* = 0.0007), and butyrate tended to be increased (Fig. 5b, c). CS exposure also affected metabolites in the TNBS-induced colitis model with a decrease in several metabolites, including glyoxylic acid and dimethylglycine (Fig. 5b). We focused on metabolites that showed opposing trends, increasing with CS exposure in the DSS-induced colitis model but decreasing in the TNBS-induced colitis model. Only two metabolites, 4-HBA and ureidopropionic acid, met these criteria, exhibiting more than a twofold increase in median levels in DSS models and a decrease to less than half in the TNBS model (Supplementary Fig. 3c). Notably, 4-HBA showed a more pronounced increases by CS exposure in the DSS colitis model (*P* = 0.011) compared to ureidopropionic acid, whereas it tended to decrease in the TNBS-induced colitis model (Fig. 5d). These findings suggest that 4-HBA produced from expanded bacteria in DSS-treated mice is associated with the severity of colitis.

### Oral administration of 4-HBA attenuates DSS-induced colitis

**Fig. 6:**
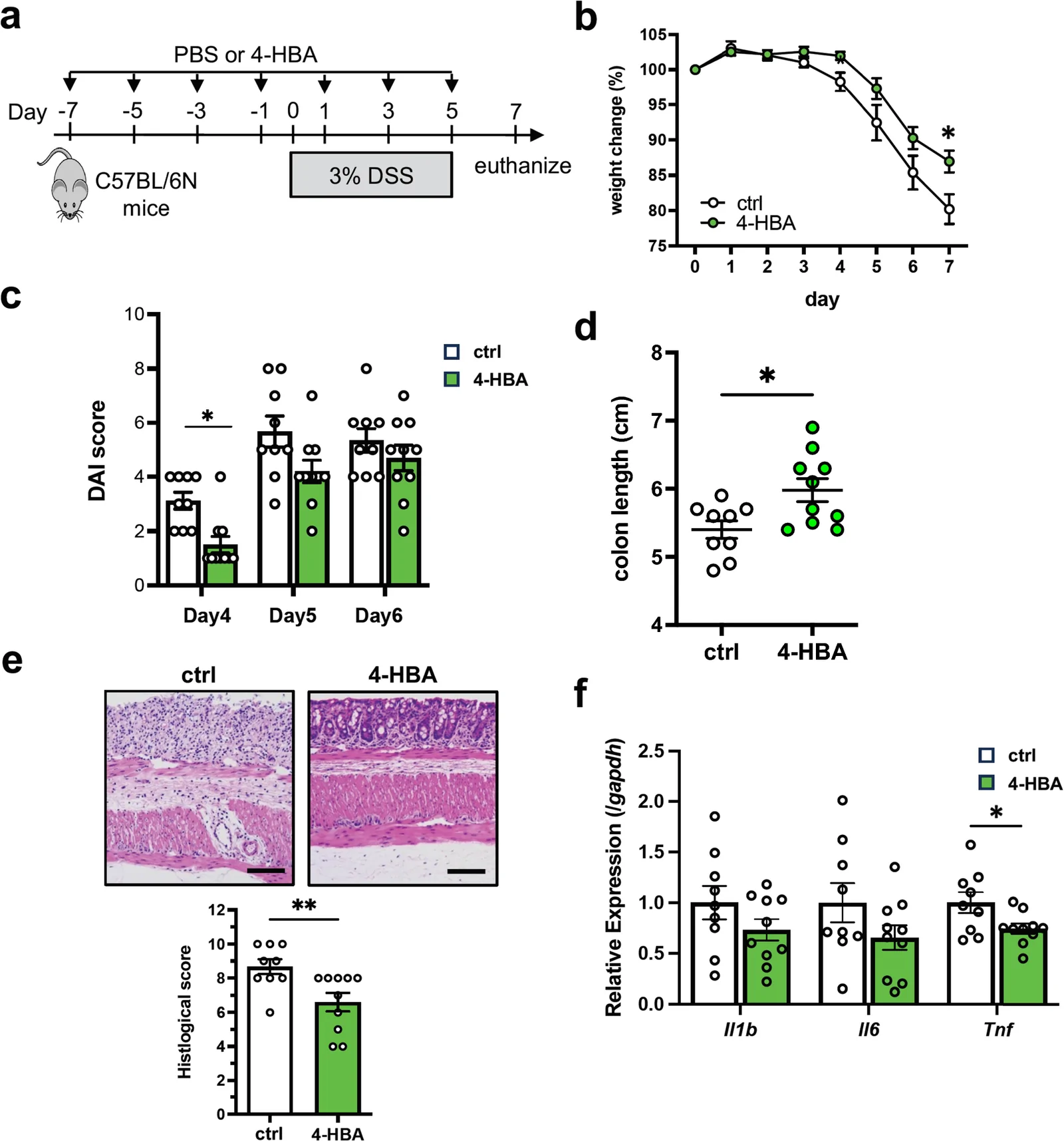
Oral administration of 4-HBA ameliorates DSS-induced colitis. **a** Schematic model of the experimental design. 4-HBA (50 mg/kg) was orally administered to mice every other day starting 7 days before DSS treatment, and then mice were administered 3% DSS in drinking water for 5 days. **b** Change in body weight in the control and 4-HBA-treated mice. **c** The DAI was monitored from days 4 to 6 after DSS administration. **d** Colon length at day 7 after DSS administration. **e** Sections of the proximal colon region were stained with hematoxylin and eosin. Representative images are shown in the upper panel, and histological scores are shown in the bottom panel. Bars 100 µm. **f** IL-1β, IL-6, and TNF-α gene expression in the colon. Data are shown as the mean ± SEM (*n* = 9–10). *P < 0.05, **P < 0.01

To investigate whether 4-HBA regulates the pathogenesis of colitis, 4-HBA was orally administered to mice before inducing colitis, followed by 3% DSS administration for 5 days (Fig. 6a). Weight loss and the DAI score at day 4 were lower in 4-HBA-administered mice compared with control mice (Fig. 6b, c). The colon length in 4-HBA-administered mice was longer than that in control mice (Fig. 6d). The pathology of colitis, including the histological score, was significantly improved in 4-HBA-administered mice (*P* = 0.0025) (Fig. 6e). An analysis of gene expression of inflammatory cytokines showed that *Tnf* levels were significantly decreased and *Il1b* and *Il6* levels tended to be decreased by 4-HBA administration (Fig. 6f). These results suggest that administration of 4-HBA ameliorates the severity of DSS-induced colitis.

**Fig. 7:**
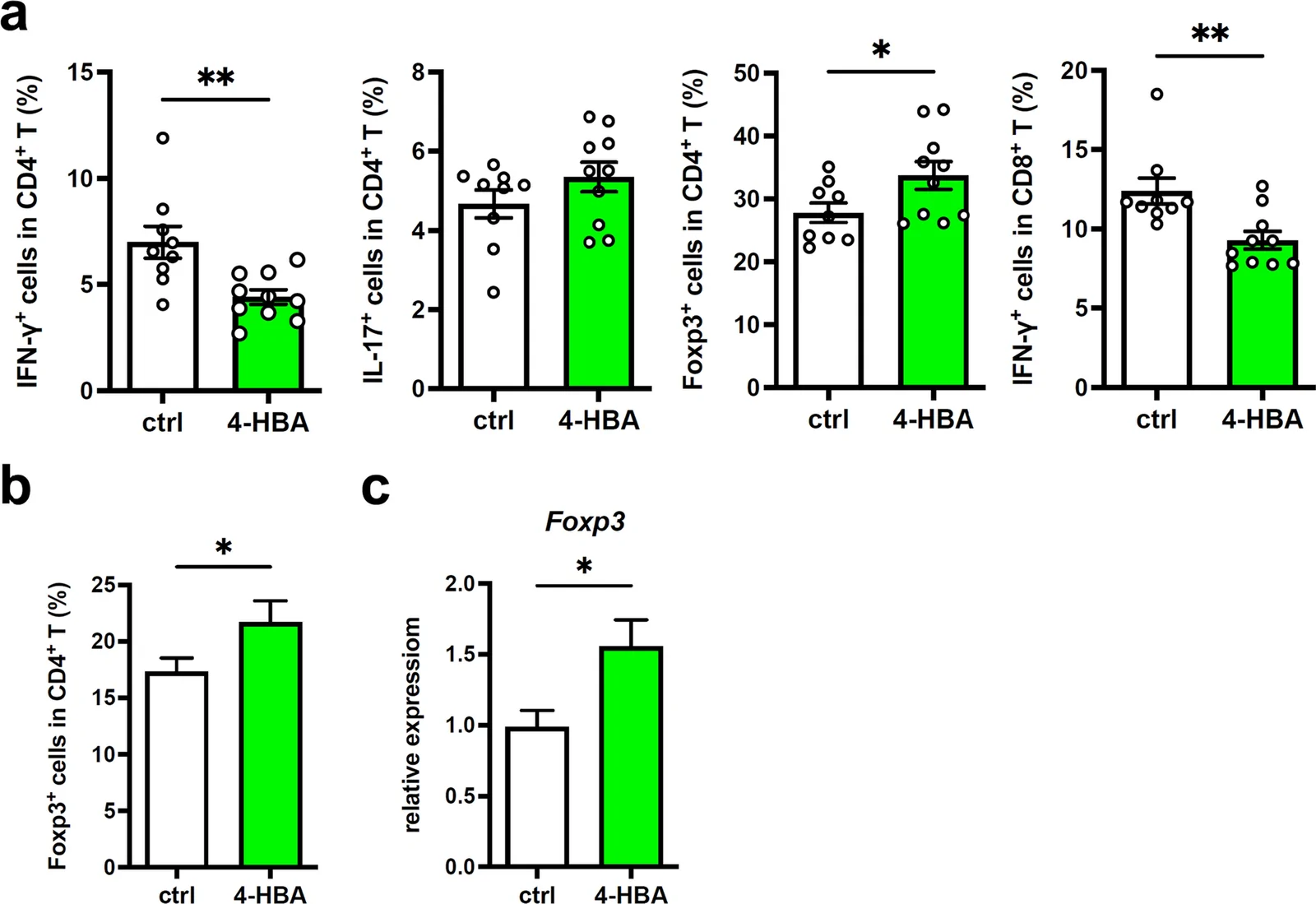
Treg differentiation is enhanced by 4-HBA. **a** Lymphocytes from the lamina propria were isolated from control and 4-HBA-administered mice in the DSS-induced colitis model at day 7 (*n* = 9–10). Proportions of Th1 cells, Th17 cells, Tregs, and IFN-γ^+^ CD8^+^ T cells, were analyzed by flow cytometry. **b**, **c** CD4^+^ T cells isolated from mesenteric lymph nodes were stimulated under Treg differentiation with or without 4-HBA (100 µg/ml). The proportion of Tregs was analyzed by flow cytometry (**b**), and gene expression of Foxp3 was analyzed by quantitative PCR (**c**). Data are shown as the mean ± SEM. *P < 0.05, **P < 0.01

We next investigated the effect of 4-HBA on effector T cells in the colon lamina propria of mice with DSS-induced colitis. The proportion of Th17 cells in 4-HBA-administered mice was similar to those in control mice. However, the proportions of Th1 cells and IFN-γ^+^ CD8^+^ T cells were significantly lower (Th1 *P* = 0.0049; IFN-γ^+^ CD8^+^ T *P* = 0.0057) and that of Tregs was significantly higher (*P* = 0.046) in 4-HBA-administered mice than in control mice (Fig. 7a). To examine whether 4-HBA directly affects Treg differentiation, CD4^+^ T cells isolated from mesenteric lymph nodes of C57BL/6N mice were cultured under the condition of Treg differentiation in the presence or absence of 4-HBA. The proportion of Foxp3^+^ cells and *Foxp3* gene expression levels were increased by 4-HBA (Fig. 7b, c). These results suggest that 4-HBA contributes to the suppression of DSS-induced colitis in part through the direct induction of Treg differentiation.

We also investigated the effect of 4-HBA on TNBS-induced colitis (Supplementary Fig. 4a). Weight loss and colon length were comparable between the control and 4-HBA-treated mice (Supplementary Fig. 4b, c). Furthermore, 4-HBA administration did not significantly improve the pathology of colon or the expression of inflammatory cytokines (Supplementary Fig. 4d, e). Consistent with no obvious efficacy, the proportions of effector T cells including Th1 cells, Th17 cells, and IFN-γ^+^ CD8^+^ T cells as well as Tregs were not significantly changed by 4-HBA administration (Supplementary Fig. 4f). These results suggested that the protective effect of 4-HBA might be affected by the type of colitis.

## Discussion

CS contains more than 4000 chemicals, including various harmful substances, such as carbon monoxide, nicotine, nitrogen oxides, and polycyclic aromatic hydrocarbons [11]. These components can be causative for various diseases, such as cancer, respiratory disease, and cardiovascular disease. Regarding IBD, CS is risk factor for CD, but it is considered a protective factor for UC [10]. To determine the underlying mechanisms of this different effect of CS, the effect of nicotine, a major component of CS, has been well studied using colitis models. Administration of nicotine attenuated the DSS-induced UC model through the increase of colonic indole levels, which stimulates the production of mucosal protective cytokine IL-22 from type 3 innate lymphoid cells [20]. Another study also showed that nicotine induced the Tregs, and suppressed Th1/Th17 response through the α7 nicotinic acetylcholine receptor-mediated signaling, leading to the attenuation of oxazolone-mediated UC models but the exacerbation of TNBS-mediated CD models [21]. In addition, ubiquinone, an aromatic compound present in CS, promoted the ectopic increase of oral bacteria in the colonic mucosa, which enhanced the Th1 response, resulting in the attenuation of oxazolone-mediated UC models but exacerbation of TNBS-mediated CD models [22]. In line with the effects of nicotine and ubiquinone on the colitis models, our study showed that CS exposure attenuated DSS-induced colitis and increased Tregs, while it exacerbated TNBS-induced colitis and increased Th1 cells. These findings suggest that different outcomes by CS exposure are at least partially attributable to the effects of these compounds, although other numerous chemicals in CS might also be involved.

The intestinal microbiota is crucial for maintaining intestinal homeostasis, and its dysbiosis is associated with the pathogenesis of IBD. Common features of dysbiosis in UC or CD patients include a considerable reduction in bacterial diversity, decreased abundance of Bacillota (formerly Firmicutes), particularly SCFA-producing bacteria (e.g., *Faecalibacterium prausnitzii*), lower intestinal SCFA levels, and an increased presence of Pseudomonadota, including *Enterobacteriaceae* [6]. CS also disrupts the microbiota balance in healthy individuals, reducing diversity and increase in Pseudomonadota [23]. Our microbiota analysis showed that CS exposure in TNBS-induced colitis, which exacerbated inflammation, significantly increased the relative abundance of *Enterobacteriaceae.* However, this increase was not observed in untreated mice, suggesting that CS components directly altered the microbiota composition, including the ectopic increase of oral bacteria, which triggered a Th1 and Th17 response that worsens the colitis. Conversely, CS exposure attenuated pathogenesis in DSS-induced colitis, coinciding with significant higher SCFA levels. SCFAs are key components that regulate gut homeostasis [24]. Mice lacking SCFA receptors, including GPR43 and GPR109a, show exacerbated chemical-induced colitis [25, 26 ]. SCFAs improve gut condition by promoting epithelial barrier function via tight junction proteins and anti-microbial peptides induction [27], suppressing *Enterobacteriaceae* proliferation [28, 29 ] and inducing colonic Treg differentiation and enhancing their suppressive activity through GPR43-mediated signaling [30, 31 ]. The observed tendency for *Bifidobacteriaceae* and *Lachnospiraceae* to increase in the CS-exposed group in DSS-induced colitis, as major SCFA-producing bacteria, likely contributed to the elevated SCFA levels and subsequent amelioration. However, the specific bacteria truly responsible for the higher SCFA levels and ameliorating the pathogenesis in the CS-exposed group remain to be identified. Therefore, fecal microbiota transplantation experiments are required to determine whether the microbiota in CS-exposed mice with DSS-induced colitis confers protection against colitis and to identify the specific bacteria associated with an improved pathogenesis.

In this study, while CS exposure induces broad alterations in various metabolites including SCFAs, which comprehensively affect the colitis models, we focused on 4-HBA. It exhibited opposing trends between the two colitis models, increasing in the DSS-induced colitis models and decreasing in the TNBS-induced colitis model, and showed a more pronounced increase in the DSS-induced colitis model compared to other candidates. In addition, we recently reported that fecal concentration of 4-HBA was increased in current smoker UC patients compared with non-smoker or former smoker UC patients [22]. Therefore, we prioritized 4-HBA as a representative metabolite to further investigate. We clearly found that oral administration of 4-HBA attenuated the DSS-induced colitis. This natural organic compound is present in various organisms, such as animals, plants, and microorganisms [32]. Its esters, known as parabens, are widely used as preservatives in pharmaceuticals and some food products, indicating that 4-HBA is a highly safe material for humans. This organic compound is an isomer of salicylic acid, which has anti-microbial, anti-inflammatory, and anti-oxidant effects [33]. In clinical practice, 5-aminosalicylic, which shares a common structural backbone with 4-HBA, is a key drug for IBD therapy, and it is effective for inducing and maintaining remission [34]. Mechanistically, 4-HBA has been shown to suppress IL-1β production by directly inhibiting the NLRP3 inflammasome and to reduce IL-1, IL-6, and TNF-α expression at the transcription level [35]. As a phenolic acid, it also exhibits anti-oxidant properties. Given that both colitis and CS induce oxidative stress, high 4-HBA concentrations might suppress the inflammation and damage. We also found that 4-HBA directly increased Treg differentiation in vitro. Therefore, 4-HBA might cooperate with SCFAs to increase Tregs and attenuate DSS-induced colitis in CS-exposed mice. In contrast, 4-HBA failed to significantly attenuate the pathogenesis of TNBS-induced colitis, though it did not lead to any worsening of the condition. Since inflammation in TNBS-induced colitis is transmural and more acute than that in DSS-induced colitis, 4-HBA was likely insufficient to overcome such a severe Th1-mediated condition, suggesting that the therapeutic efficacy of 4-HBA might depend on the specific type of colitis and its distinct pathophysiological mechanisms. Furthermore, 4-HBA concentrations were not elevated by CS exposure in untreated mice, which suggested that 4-HBA was synthesized by intestinal microbes during DSS-induced colitis. This compound is synthesized from chorismate via the shikimate pathway in bacteria and used for ubiquinone synthesis. Whether 4-HBA is contained in CS components is unknown. Therefore, further investigation is required to determine what types of bacteria synthesize 4-HBA.

The present study has several limitations. First, to investigate the effect of CS in IBD models, we used two types of chemical-induced colitis, DSS-induced and TNBS-induced colitis, in female C57BL/6N mice. Although these experimental colitis models induced inflammation of the superficial mucosa and transmural inflammation, respectively, they do not fully represent the complex pathogenesis of human UC and CD. Specifically, these models primarily elicit acute inflammation, and our investigation of CS and effects of 4-HBA focused on this early phase of inflammation. Additionally, mouse strain and sex differences affect the susceptibility of colitis, although there is no sex difference in the development of IBD. Therefore, other chronic inflammation models, such as IL-10 deficient mice [36] or T-cell adoptive transfer models [37], using mice with various genetic backgrounds and different sexes, might be necessary for a more comprehensive evaluation of the effects of CS and 4-HBA. Second, we did not directly quantify the intensity of CS exposure, such as nicotine or carbon monoxide levels. Since we utilized high-yield commercial cigarettes, the precise chemical yield and biological dose may vary compared to standardized research cigarettes such as 3R4F. In addition, our protocol involved a condensed, high-intensity exposure (20 cigarettes within 30 min), which differs from typical human smoking patterns. However, the exposure quantity in our model is comparable to, or even more intensive than, previously established CS-exposure models [38, 39 ]. In line with those studies, we observed the attenuation of inflammation in the UC-like model and its exacerbation in the CD-like model, reflecting the clinical observation of smoking’s divergent effects in UC and CD. Third, to assess the effect of CS on the immune response in colitis models, our analysis was limited to effector T cells. Innate immune responses and epithelial cell damage also considerably contribute to the pathogenesis of colitis, particularly in DSS-induced models, which induce robust infiltration of neutrophils. Fourth, each analysis of effector T-cell populations, the composition of the microbiota, and intestinal metabolites in CS-exposed mice was performed in distinct sets of mice. Consequently, we could not show a direct association between immune cells, the microbiota, and metabolites.

In summary, this study shows that CS exposure attenuates the pathogenesis of DSS-induced colitis, while it exacerbates TNBS-induced colitis. This differential effect of CS exposure is associated with changes in the composition of the microbiota, intestinal metabolites, and immune responses. We also identified a novel mechanism underlying the CS-mediated modulation of colitis, demonstrating that the CS-induced increase in the intestinal metabolite 4-HBA functions as a protective factor through the induction of Tregs. These mechanistic findings identify 4-HBA as a potential therapeutic target and provide a foundation for developing new therapeutic approaches for UC.

## Supplementary Information

Below is the link to the electronic supplementary material.

Supplementary file1 (PDF 5454 KB)

## Abbreviations

IBD: Inflammatory bowel disease
UC: Ulcerative colitis
CD: Crohn’s disease
CS: Cigarette smoking
DSS: Dextran sodium sulfate
TNBS: 2,4,6-Trinitrobenzene sulfonic acid
4-HBA: 4-Hydroxybenzoic acid
Treg: Regulatory T cell
Th: T helper type
IL: Interleukin
DAI: Disease activity index
LPL: Lamina propria lymphocyte
SCFA: Short-chain fatty acid
