# Supplementary material for: Metabolite 4-hydroxybenzoic acid, modulated by cigarette smoke exposure, functions as a protective factor in dextran sodium sulfate-induced colitis
Source: J Gastroenterol. 2026 Jul 31;61(11):1593–605. doi: 10.1007/s00535-026-02484-4 (PMC13695684; doi:10.1007/s00535-026-02484-4)
Supplement: Supplementary file 1 [file 535_2026_2484_MOESM1_ESM.pdf]

Supplemental information

Metabolite 4-hydroxybenzoic acid, modulated by cigarette smoke exposure, functions as a protective factor in dextran sodium sulfate-induced colitis

Short title: 4-HBA protects experimental UC

Masahiro Kitabatake[1], Tatsuki Nishioka[1], Atsushi Hara[1], Kaito Yasuike[1], Yumiko Nakanishi[2], Eiji Miyauchi[2], Noriko Ouji-Sageshima[1], Hiroshi Ohno[2], and Toshihiro Ito[1]

[1]Department of Immunology, Nara Medical University, Kashihara, Japan

[2]Laboratory for Intestinal Ecosystem, RIKEN Center for Integrative Medical Sciences, Yokohama, Japan

Corresponding author: Toshihiro Ito, M.D., Ph.D.

Department of Immunology, Nara Medical University, 840 Shijo-cho, Kashihara, Nara, 634-8521, Japan.

Tel: +81-744-22-3051; fax: +81-744-29-7503; e-mail: toshi-ito@naramed-u.ac.jp

Methods

Mice

Female C57BL/6N mice (7 weeks old) were purchased from CLEA Japan (Tokyo, Japan), and maintained under specific pathogen-free conditions at the Animal Research Center, Nara Medial University. All procedures were carried out in accordance with the regulations for animal care. The experimental protocols were approved by the Ethics Review Committee for Animal Experimentation of Nara Medical University (approval number: 12232) and the Animal Research: Reporting of In Vivo Experiments (ARRIVE) guideline. The details methods are provided in supplemental information.

CS exposure

Non-filtered cigarettes, containing nicotine 2.3 mg and tar 28 mg, were purchased from Japan Tabaco Inc. (Peace®; Tokyo, Japan). Mice were exposed to smoke from 20 cigarettes for 30 min, 6 days a week using an exposure apparatus (MIPS Inc., Osaka, Japan) [1]. Smoke was generated by inhaling a fired cigarette with a compressor in the apparatus, and then mixed with seven volumes of air. The mice, which were separately kept in holders connected to the apparatus, were exposed to smoke for 2 s, followed by air for 2 s, via their noses at a rate of 15 puffs/min. Mice in control group were also kept for 30 min in the apparatus holders without CS exposure.

Colitis models

DSS (molecular weight: 36,000–50,000 Da, colitis grade) was purchased from MP Biomedicals (Santa Ana, CA). At 2 weeks after initiating CS exposure, the mice were administrated 3% DSS in drinking water *ad libitum* for 5 days, followed by tap water for

2 days. Disease activity was evaluated using the disease activity index (DAI), as previously described [2]. The DAI score was calculated form the sum of scores for weight loss (0 = < 1%, 1 = 1%–5%, 2 = 5%–10%, and 3 = > 10%), stool consistency (0 = normal pellets, 1 = soft pellets without adhering to the anus, 2 = very soft pellets adhering to the anus, and 3 = liquid stool on long streams), and rectal bleeding (0 = none, 1 = small spots of blood in the stool with a dry anal region, 2 = large spots of blood in the stool and blood appears through the anal orifice, 3 = deep red stool and blood spreads largely around the anus) In some experiments, 4-HBA (Sigma-Aldrich, St Louis, MO) was orally administered at 50 mg/kg in mice every other day from 7 days before starting DSS administration. In another model of colitis, following overnight fasting, mice were intrarectally administrated 3 mg of TNBS (Tokyo Chemical Industry, Tokyo, Japan) dissolved in 100 µl of 50% ethanol using a vinyl catheter that was inserted 3.5 cm from the anus under anesthetization with sodium pentobarbital (50 mg/kg; Fujifilm Wako) or a combination of medetomidine (0.75 mg/kg, Nippon Zenyaku Kogyo, Fukushima, Japan), midazolam (4 mg/kg, Maruishi Pharmaceutical, Osaka Japan), and butorphanol (5 mg/kg, Meiji Animal Health, Tokyo, Japan). After TNBS injection, the mice were kept in the head down position for 5 min. The body weight was measured daily, and the mice were euthanized and analyzed 3 days after the induction of colitis. In some experiments, 4-HBA (50 mg/kg) was orally administered every other day from 7 days before TNBS administration, followed by three additional doses after colitis induction.

Histological analysis

The large intestines were surgically excised, their length was measured, and then they were longitudinally cut. Pieces of the rectal side were fixed with 4%

paraformaldehyde. After embedding in paraffin, 1–2-μm sections were cut and then stained with hematoxylin and eosin (Sakura Finetek Japan Co., Ltd., Tokyo, Japan). The pathogenesis of colitis was assessed by two independent pathologists according to the histological score, as previously described [3]. The histological score was the sum of scores for the severity of inflammation (0 = no inflammation, 1 = mild, 2 = moderate, and 3 = severe), thickness of inflammatory involvement (0 = no inflammation, 1 = mucosa, 2 = mucosa plus submucosa, and 3 = transmural), epithelial damage (0 = intact epithelium, 1 = disruption of architectural structure, 2 = erosion, and 3 = ulceration), and extent of damage to lesions (0 = no lesions, 1 = punctuate, 2 = multifocal, and 3 = diffuse).

Quantitative polymerase chain reaction

Total RNA was purified from the distal region of the colon by the NucleoSpin RNA Kit (Macherey-Nagel GmbH, Duren, Germany), and then complementary DNA was generated by Superscript IV reverse-transcriptase (Thermo Fisher Scientific, Waltham, MA). Quantitative polymerase chain reaction (PCR) was performed using Taqman gene expression assays (*Gapdh*, Mm99999915; *Il1b*, Mm00434228; *Il6*, Mm00446190; *Tnf*, Mm00443258; *Foxp3*, Mm00475162) on StepOne Plus (Thermo Fisher Scientific) using default cycling conditions. Gene expression was analyzed by the relative ΔΔCT method and normalized to *Gapdh* expression.

Flow cytometry

Lymphocytes from the lamina propria in the colon of colitis-induced mice were isolated by using the Lamina Propria Dissociation Kit with the gentleMACS dissociator (Miltenyi Biotec GmbH, Bergisch Gladbach, Germany), as described previously [4].

Briefly, to obtain a single-cell suspension, colon pieces were pre-incubated with Hank's balanced salt solution (HBSS) containing 0.1% bovine serum albumin, 1 mM dithiothreitol, and 5 mM EDTA, and then digested with the Lamina Propria Dissociation Kit and gentleMACS dissociator. The cells were stained with Zombie-Aqua or Zombie-NIR (Biolegend, San Diego, CA) to exclude dead cells from the analysis. The cells were reacted with monoclonal antibodies against surface antigens and Fc receptor blocker (eBioscience, San Diego, CA). The following antibodies (purchased from Biolegend) were used for cell surface staining: BV421-labeled anti-CD45 (30-F11), fluorescein isothiocyanate (FITC)-labeled and allophycocyanin (APC)-labeled anti-CD4 (GK1.5), phycoerythrin (PE)-labeled anti-CD25 (PC61), PE-Cy7-labeled anti-CD3 (145-2C11), and APC-Cy7-labeled anti-CD8a (53-6.7). To detect Foxp3, the cells were fixed and permeabilized with the Mouse Foxp3 Buffer Set (BD Biosciences, San Jose, CA) and then stained with AlexaFluor 647-labeled anti-Foxp3 (MF23; BD Biosciences). To perform intracellular cytokine staining, the cells were stimulated with 50 ng/ml of phorbol 12-myristate 13-acetate (Sigma-Aldrich) and 500 ng/ml of ionomycin (Sigma-Aldrich) in the presence of GolgiStop (BD Biosciences) for 4 h. After stimulation, the cells were stained with the antibodies for surface antigen, and intracellular cytokines were stained with FITC-labeled anti-interferon (IFN)-γ (11-7311; Biolegend) and PE-labeled anti-IL-17A (Biolegend). The stained cells were analyzed using the MACSQuant Analyzer (Miltenyi Biotec GmbH) or the Northern Lights spectral flow cytometer (Cytek Biosciences, Fremont, CA), and data were analyzed by FlowJo software (Tree Star, Ashland, OR), and gating strategies for the detection of these cell populations are shown in **Supplementary Figure 1**.

In vitro Treg differentiation

CD4+ T cells were isolated from mesenteric lymph nodes of C57BL/6N mice using a CD4+ T cell isolation kit and autoMACS separator (Miltenyi Biotec GmbH) according to the manufacturer's instructions. The cells were stimulated with coated anti-CD3 mAb (0.5 μg/ml, Biolegend) and soluble factors, including anti-CD28 mAb (0.5 μg/ml; Biolegend), murine IL-2 (10 ng/ml; Peprotech, Cranbury, NJ), and human TGF-β (5 ng/ml; Peprotech), in the presence or absence of 4-HBA (100 μg/ml) for 48 h. After the stimulation, Treg differentiation was evaluated by measuring Foxp3 expression using flow cytometry and quantitative PCR, as described above.

Microbiota analysis

Fecal samples were obtained and then immediately frozen by dry ice. Extraction of bacterial DNA, sequencing, and software analysis were carried out by Morinaga Milk Industry (Shizuoka, Japan), as described previously [4]. Briefly, fecal samples (20 mg) from the mice were crushed in saturated phenol with glass beads and the Multi-Beads Shocker (Yasui Kikai Co., Osaka, Japan), and then bacterial DNA was extracted by phenol/chloroform and isopropanol precipitation. The V3–V4 region of the bacterial 16S ribosomal RNA gene was amplified by PCR using the following primer set: Tru357F (5′-CGCTCTTCCGATCTCTGTACGGRAGGCAGCAG-3′) and Tru806R (5′-CGCTCTTCCGATCTGACGGACTACHVGGGTWTCTAAT-3′). The PCR product was further amplified using the barcoded primers adapted for the Illumina MiSeq system (Illumina Inc., San Diego, CA). After purification by the QIAquick PCR Purification Kit (Qiagen, Valencia, CA), equal amounts of amplicons were pooled and purified with the GeneRead Size Selection Kit (Qiagen). The pooled libraries were sequenced by the

Illumina Miseq system with the MiSeq Reagent Kit v3. Sequence data were analyzed using the QIIME software package version 1.8.0 (http://qiime.org/) after removal of phiX reads from raw paired-end reads. Potential chimeric sequences were removed by UCHIME, and then operational taxonomic units were assigned by open-reference operational taxonomic unit picking with a 97% threshold of pairwise identity. All sequences were used for the taxonomical classification performed using the Greengenes reference database (gg_13_5_99) (http://greengenes.secondgenome.com/). Equal numbers of sequences were used for the analysis of alpha-diversity indices, the chao1 index and the Shannon index, which were calculated by QIIME software version 1.8.0. Linear regression analysis was performed on the relative abundance of gut microbiota at the family level using the R package MaAsLin2, with the parameters normalization = "NONE" and transform = "AST". Subsequently, for visualization, the -log10-transformed q-values were multiplied by the sign of the corresponding coefficients and then plotted.

Metabolite analysis

An analysis of intestinal metabolites in feces was conducted as described elsewhere [5]. Fecal samples were extracted separately for the analysis of water-soluble metabolites and short-chain fatty acids (SCFAs). The extracts were subjected to derivatization and subsequently analyzed by gas chromatography-tandem mass spectrometry platform on a GCMS-TQ8030 (Shimadzu, Kyoto, Japan). The data were processed using LabSolution Insight (Shimadzu, Kyoto, Japan).

Statistical analysis

Data are presented as the mean ± standard error of the mean (SEM) and are representative of at least two independent experiments. Student's *t*-test was used to perform statistical analyses of body weight changes, colon length, gene expression, metabolites levels and lymphocyte populations between the control and CS-exposed group using GraphPad Prism 9 (GraphPad Software, San Deigo, CA). Statistical analysis of metabolites levels across all models was performed by one-way analysis of variance with Tukey's post-hoc test. For DAI, and histological scores, the Mann–Whitney *U*-test was used. Differences were considered statistically significant at $p < 0.05$.

Reference

1. Tomoda K, Kubo K, Asahara T, et al. Cigarette smoke decreases organic acids levels and population of bifidobacterium in the caecum of rats. J Toxicol Sci. 2011;36:261-266.
2. Gommeaux J, Cano C, Garcia S, et al. Colitis and colitis-associated cancer are exacerbated in mice deficient for tumor protein 53-induced nuclear protein 1. Mol Cell Biol. 2007;27:2215-2228.
3. Hudert CA, Weylandt KH, Lu Y, et al. Transgenic mice rich in endogenous omega-3 fatty acids are protected from colitis. Proc Natl Acad Sci U S A. 2006;103:11276-11281.
4. Terada-Ikeda C, Kitabatake M, Hiraku A, et al. Maternal supplementation with Bifidobacterium breve M-16V prevents their offspring from allergic airway inflammation accelerated by the prenatal exposure to an air pollutant aerosol. PLoS One. 2020;15:e0238923.
5. Takeuchi T, Kameyama K, Miyauchi E, et al. Fatty acid overproduction by gut commensal microbiota exacerbates obesity. Cell Metab. 2023;35:361-375 e9.

Supplementary Fig. 1

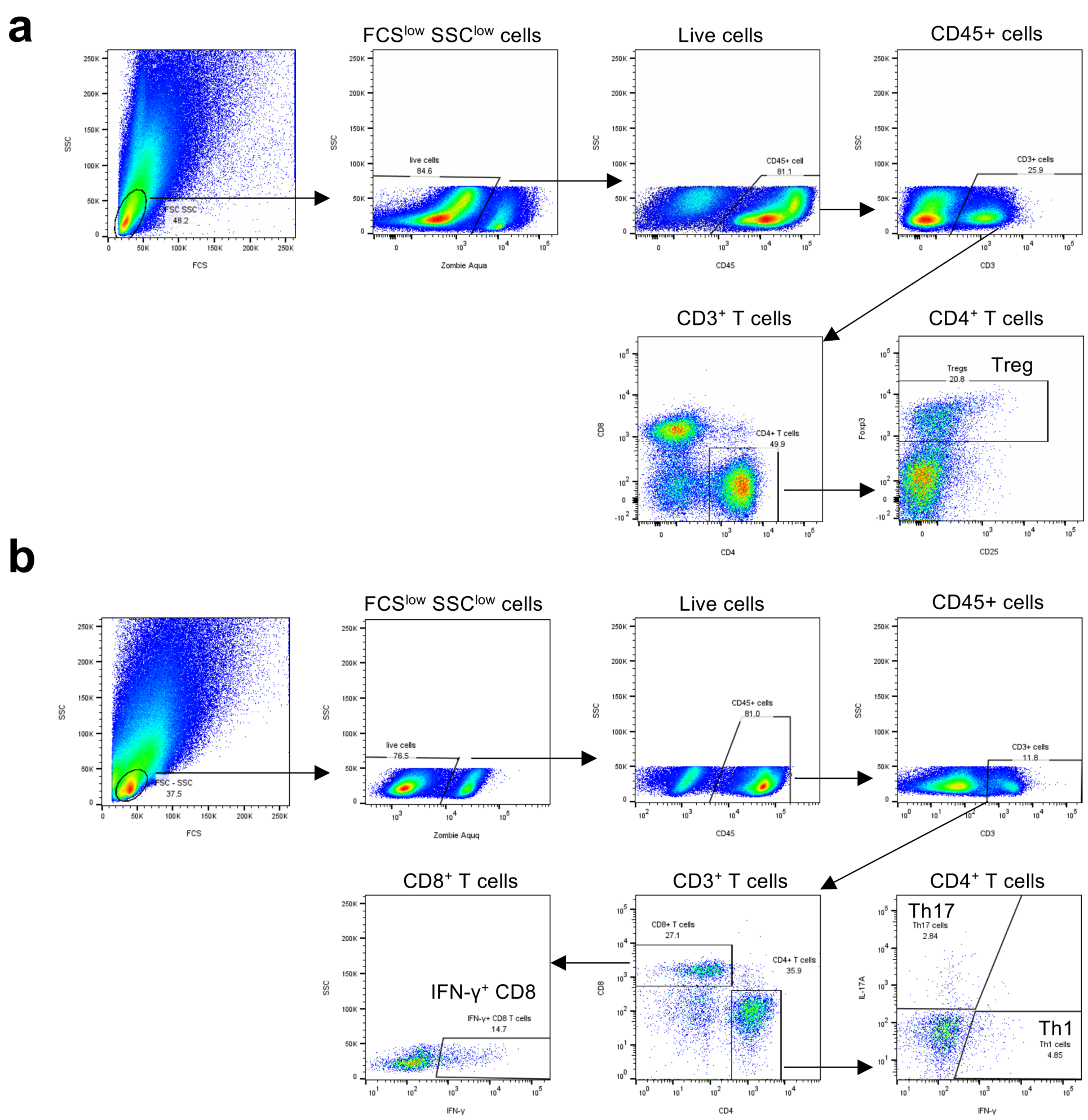

Gating strategies for the detection of Tregs, Th1, Th17 and IFN-γ$^+$ CD8$^+$ T cells in lamina propria lymphocytes by flow cytometry. (**a**) For the detection of Treg, lamina propria lymphocytes (LPLs) were stained with Zombie-Aqua to exclude dead cells, followed by surface antigen staining and intracellular staining for Foxp3. Gating was first applied to the lymphocyte population based on low forward and side scatter, followed by exclusion of dead cells using the Zombie Aqua-negative fraction. Tregs were defined as Foxp3$^+$ cells within CD45$^+$ leukocytes, gated sequentially through CD3$^+$ T cells, and then the CD4$^+$ subset. (**b**) For cytokine detection, LPLs were stimulated with PMA and ionomycin in the presence of GolgiStop for 4 hours prior to staining. Gaiting was performed in the same manner as described above, and Th1 and Th17 cells were defined as IFN-γ$^+$ and IL-17A$^+$ cells, respectively, within the CD4$^+$ T cell population.

Supplementary Fig. 2

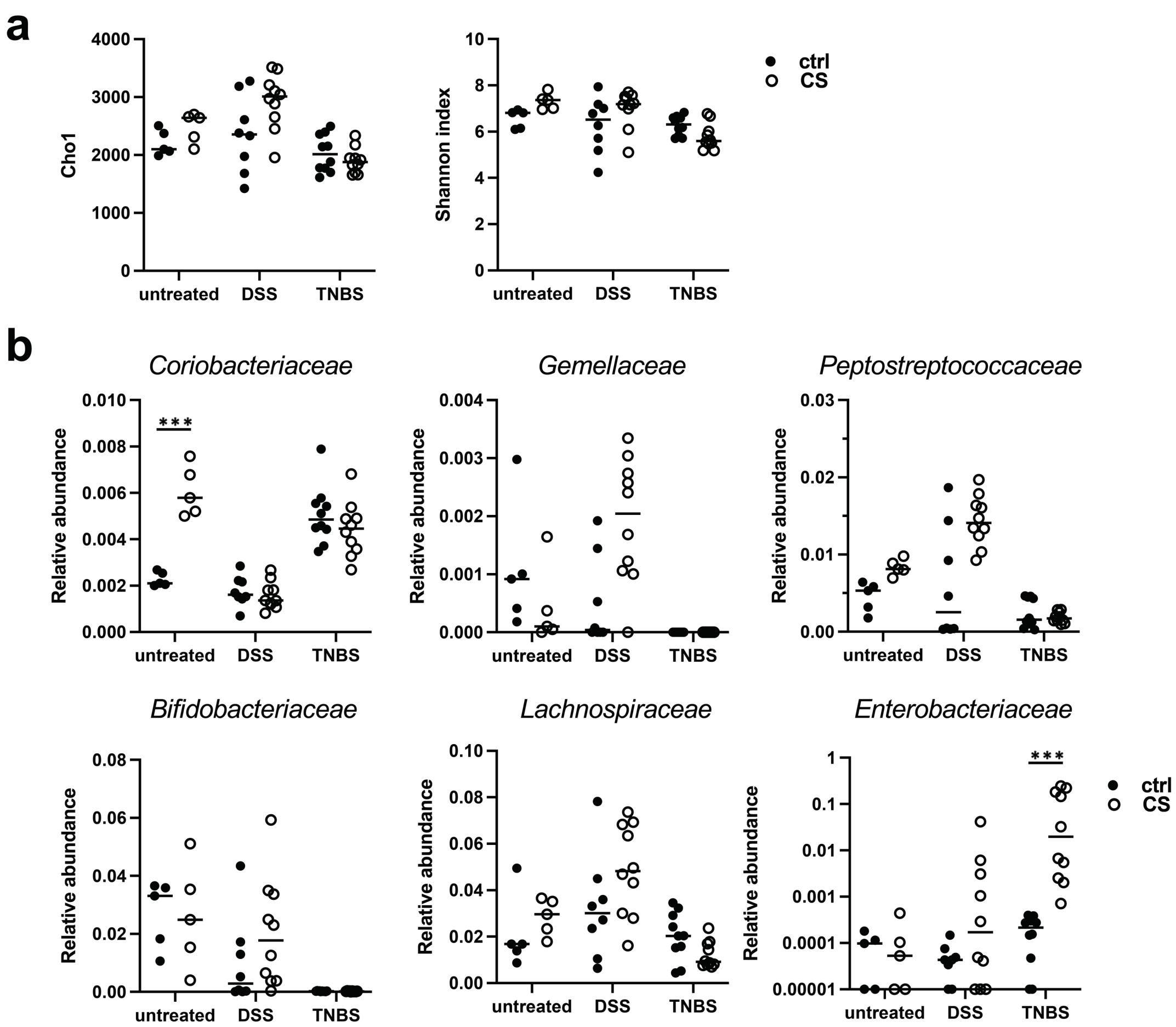

Composition of the gut microbiota in untreated, DSS-treated, and TNBS-treated mice in control and CS-exposed group. Compositions of fecal bacteria of control and CS-exposed group from untreated, DSS-treated (day 7) and TNBS-treated mice (day 3) were analyzed by 16S ribosomal RNA gene sequencing (**a**) Alpha-diversity indices, chao1 index and Shannon index, in each mice were plotted. (**b**) The relative abundance of *Coriobacteriaceae*, *Gemellaceae*, *Peptostreptococcaceae*, *Bifidobacteriaceae*, *Lachnospiraceae*, and *Enterobacteriaceae*. Data are shown as the mean ± SEM with individual plots. Untreated mice (n = 5, each) DSS-treated and TNBS-treated mice (n=9-10). *** $p < 0.001$

Supplementary Fig. 3

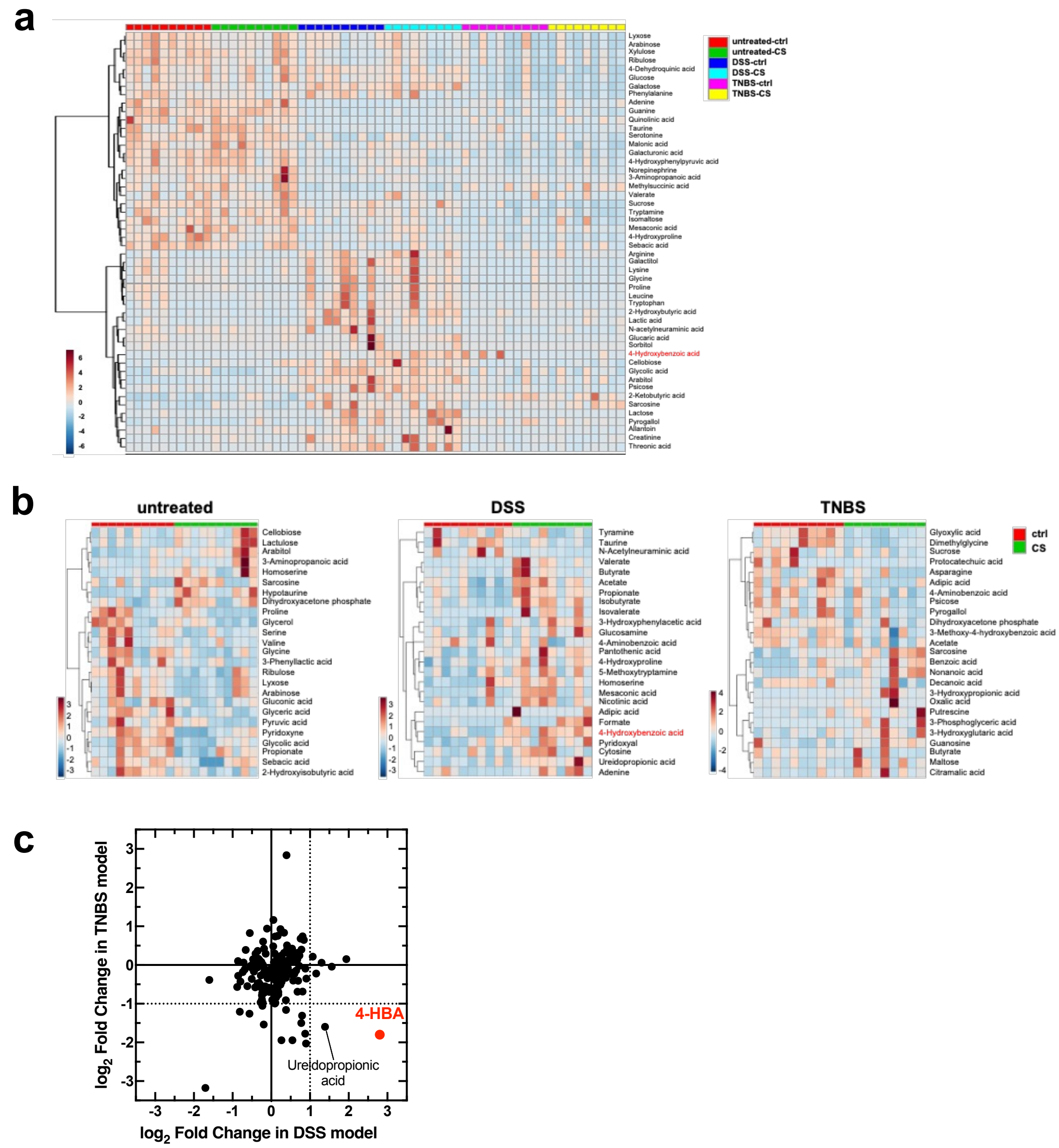

Composition of intestinal metabolites in untreated, DSS-treated, and TNBS-treated mice in control and CS-exposed group. Feces were collected from control and CS-exposed group from untreated, DSS-treated (day 7) and TNBS-treated mice (day 3). (**a**) Heat map of top 50 metabolites that were differentially expressed across all samples. (**b**) Heat map of top 25 metabolites with CS-related differences within individual models, untreated (left), DSS-treated (mid), and TNBS-treated model (right) (**c**) Scatter plot comparing metabolic changes induced by CS-exposure. Fold changes were calculated based on the median values of each group in the DSS and TNBS models. Each dot represents an individual metabolite. Dashed lines indicate the thresholds for selection: 2-fold increase in the DSS model and 50% reduction in the TNBS model (n=9-10).

Supplementary Fig. 4

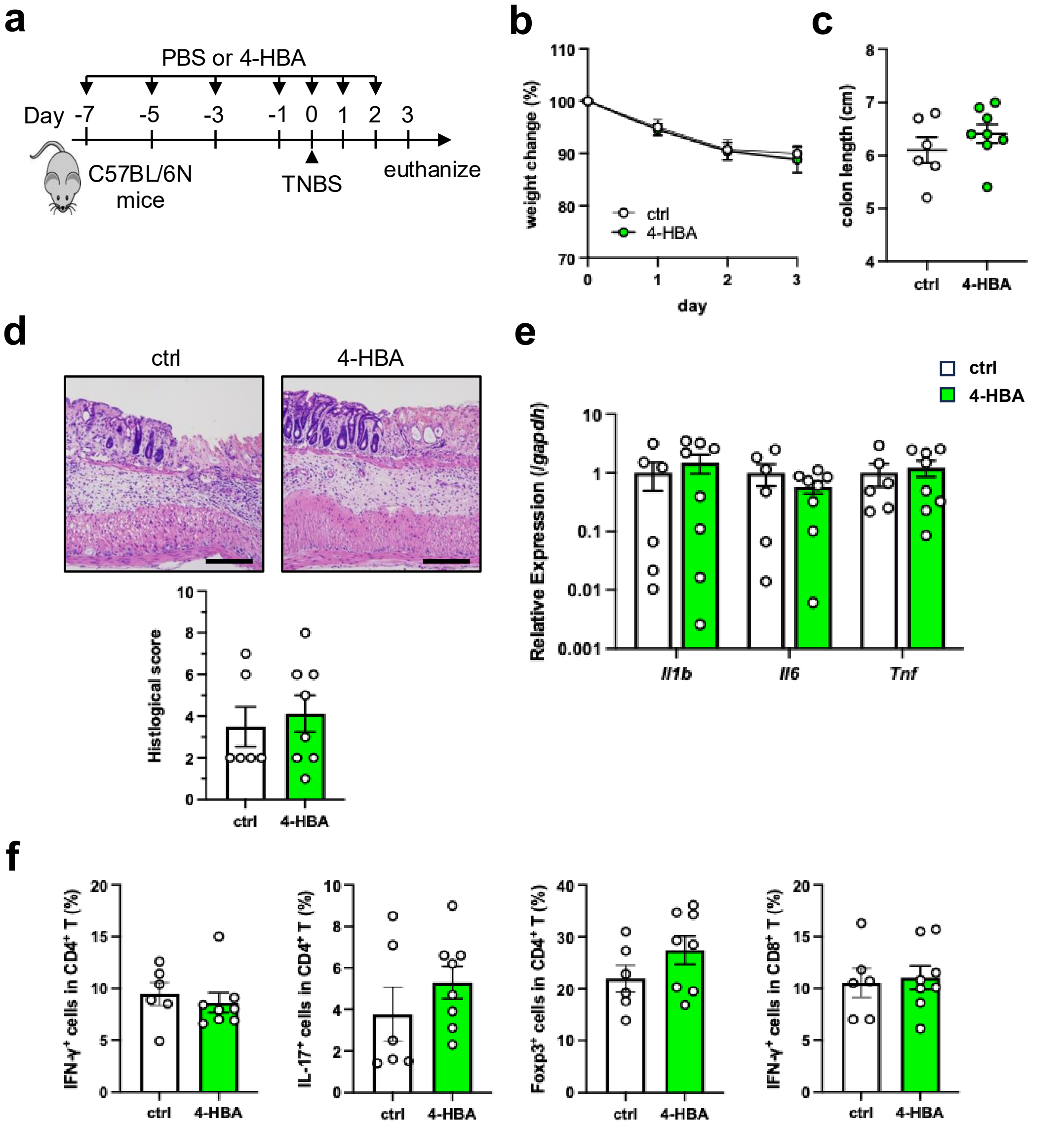

Effect of 4-HBA on TNBS-induced colitis. (**a**) Schematic model of the experimental design. 4-HBA (50 mg/kg) was orally treated to mice every other day from 7 days before TNBS administration, followed by three additional doses after colitis induction. (**b**) Change in body weight in the control and 4-HBA-treated mice. (**c**) Colon length at day 3 after TNBS administration. (**d**) Sections of the proximal colon region were stained with hematoxylin and eosin. Representative images are shown in the upper panel, and histological scores are shown in the bottom panel. Bars 100 μm. (**e**) IL-1β, IL-6, and TNF-α gene expression in the colon. (**f**) Proportions of Th1 cells (IFN-γ^+ CD4$^+$ T), Th17 cells (IL-17A$^+$ CD4$^+$ T), Tregs (Foxp3$^+$ CD4$^+$ T), and activated CD8$^+$ T cells (IFN-γ+ CD8$^+$ T), were analyzed by flow cytometry. Data are shown as the mean ± SEM with individual plots (n = 6-8).
